# A proteomics approach to identifying novel protein targets involved in erinacine A–mediated inhibition of colorectal cancer cells’ aggressiveness

**DOI:** 10.1111/jcmm.13004

**Published:** 2016-10-06

**Authors:** Ko‐Chao Lee, Hsing‐Chun Kuo, Chien‐Heng Shen, Chien‐Chang Lu, Wen‐Shih Huang, Meng‐Chiao Hsieh, Cheng‐Yi Huang, Yi‐Hung Kuo, Yung‐Yu Hsieh, Chih‐Chuan Teng, Li‐Ya Lee, Shui‐Yi Tung

**Affiliations:** ^1^ Division of Colorectal SurgeryDepartment of SurgeryChang Gung Memorial HospitalKaohsiung Medical CenterChang Gung University College of MedicineKaohsiungTaiwan; ^2^ Department of NursingChang Gung University of Science and TechnologyChiayiTaiwan; ^3^ Research Center for Industry of Human EcologyChang Gung University of Science and TechnologyTaoyuanTaiwan; ^4^ Chronic Diseases and Health Promotion Research CenterCGUSTChiayiTaiwan; ^5^ Department of Hepato‐GastroenterologyChang Gung Memorial HospitalChiayiTaiwan; ^6^ Graduate Institute of Clinical Medical SciencesCollege of MedicineChang Gung UniversityTaoyuanTaiwan; ^7^ Division of Colon and Rectal SurgeryDepartment of SurgeryChang Gung Memorial Hospital ChiayiChiayiTaiwan; ^8^ Chang Gung University College of MedicineTaoyuanTaiwan; ^9^ Grape King Biotechnology Inc (Grape King Bio Ltd.)Zhong‑LiTaiwan

**Keywords:** erinacine A, ROS, PI3K, mTOR, p70S6K, COFL1, PROF1

## Abstract

Erinacine A, a major active component of a diterpenoid derivative isolated from *Hericium erinaceus* mycelium, has been demonstrated to exert anticancer effects. Herein, we present an investigation of the molecular mechanism of erinacine A induction associated with cancer cells’ aggressive status and death. A proteomic approach was used to purify and identify the differentially expressed proteins following erinacine A treatment and the mechanism of its action in apoptotic and the targets of erinacine A. Our results demonstrate that erinacine A treatment of HCT‐116 and DLD‐1 cells increased cell cytotoxicity and reactive oxygen species (ROS) production as well as decreased cell proliferation and invasiveness. Ten differentially displayed proteins were determined and validated *in vitro* and *in vivo* between the erinacine A‐treated and untreated groups. In addition, erinacine A time‐dependent induction of cell death and inhibitory invasiveness was associated with sustained phosphorylation of the PI3K/mTOR/p70S6K and ROCK1/LIMK2/Cofilin pathways. Furthermore, we demonstrated that erinacine A–induced HCT‐116 and DLD‐1 cells viability and anti‐invasion properties by up‐regulating the activation of PI3K/mTOR/p70S6K and production of ROS. Experiments involving specific inhibitors demonstrated that the differential expression of cofilin‐1 (COFL1) and profilin‐1 (PROF1) during erinacine A treatment could be involved in the mechanisms of HCT‐116 and DLD‐1 cells death and decreased aggressiveness, which occurred *via* ROCK1/LIMK2/Cofilin expression, with activation of the PI3K/mTOR/p70S6K signalling pathway. These findings elucidate the mechanism of erinacine A inhibiting the aggressive status of cells by activating PI3K/mTOR/p70S6K downstream signalling and the novel protein targets COF1 and PROF1; this could be a good molecular strategy to limit the aggressiveness of CRC cells.

## Introduction

**Figure 1:**
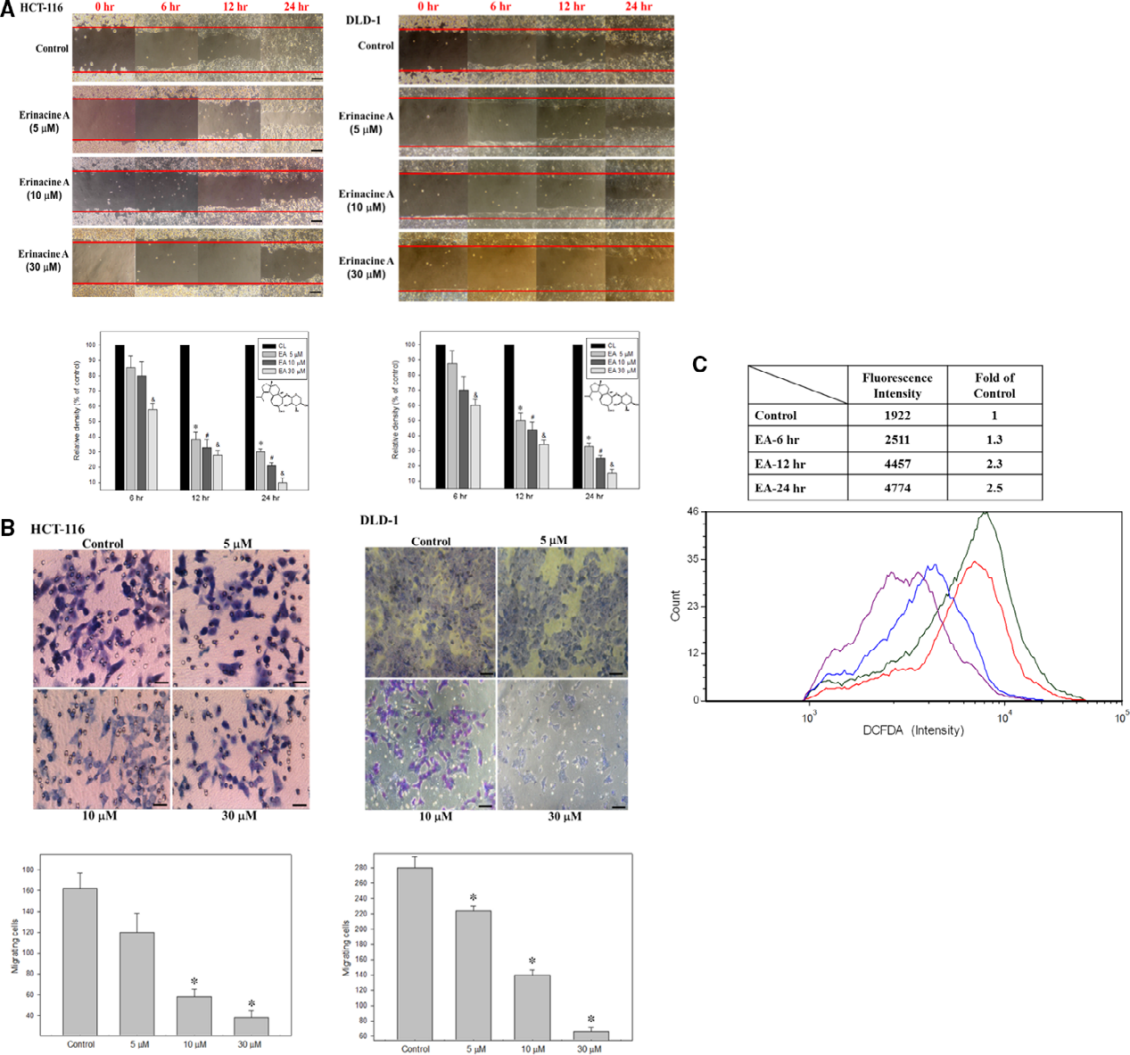
Effects of erinacine A on *in vitro* cell migration and invasiveness of human colorectal cancer cells. (**A**) HCT‐116 and DLD‐1 cells were incubated with erinacines A for 6, 12 and 24 hrs, and the migration using the scratch‐wound assay was visualized as described in Materials and methods. The percentage of surface area filled by the HCT‐116 cells was subsequently quantified by densitometric analyses relative to the control, which was set at 100% in the graph. Data are presented as means ± S.D. based on three independent experiments. The experiments were performed in triplicate, and data are presented as means ± S.D. *P < 0.05, compared with the control group for 6 hrs. #*P* < 0.05, compared with the control group for 12 hrs. &*P* < 0.05, compared with the control group for 24 hrs. (**B**) Effect of erinacine A on invasiveness of HCT‐116 and DLD‐1 cells. Cells were incubated with various concentrations of erinacine A for 24 hrs. Invasion through a layer of matrigel was determined by a Boyden Chamber method as described in ‘Materials and methods’. The lower and upper chemotaxis cells were separated by a polycarbonate membrane. Microscopy images detected cells that migrated into the inner membrane, magnification: ×200. The cell migration was quantified by counting the number of cells that migrated into the inner membrane. Control cells remained untreated. The experiments were performed in triplicate, and data are presented as means ± S.D. The symbol * indicates means that are significantly different when compared to the control group with *P* < 0.05, respectively. (**C**) HCT‐116 cells were treated with erinacine A for the indicated times, and intracellular ROS were determined by FACS analysis as described in ‘Materials and methods’. Representative histograms showed typical H2DCFDA profiles. The production of ROS was expressed as the fold of the control group.

*Hericium erinaceus*, an edible mushroom with medicinal properties, is generally a good source of nutrients and health‐promoting compounds. It is used as the culinary and medicinal *H. erinaceus*, known as Yamabushitake or Lion's Mane in Japan and China, without harmful effects 1. *Hericium erinaceus* fruit bodies and mycelium contain a large number of structurally different components with valuable biological properties 2. Either the mycelium (erinacines A‐I) or the fruit bodies (Hericenone C‐H) are the source of many bioactive extracts with drug efficacy 3. Numerous studies have suggested that *H. erinaceus* possesses a number of therapeutic properties, such as antioxidant activity 1, hypolipidemic activity 4, haemagglutinating activity 5, antimicrobial activity 6, antiaging activity 7 and immune modulation and anticancer activities 8, 9. Erinacine A (Fig. 1) collected by Chen *et al*. has been isolated from the mycelium of *H. erinaceus* in Taiwan and found to have anti‐inflammation and anticancer effects 10, 11, 12. In addition, our previous study showed that *H. erinaceus* mycelium and extracted erinacine A could be used to investigate *in vitro* and *in vivo* antitumour activity through cell cycle arrest in the G1 phase of human DLD‐1 cancer cells involved in the generation of the ROS activates p70S6K, mitogen‐activated protein kinases (MAPK) and NF‐kB pathways, which leads to p21 expression and cdk2/cyclin E and cdk4/cyclin D1 inactivation 12. However, little is known about the anti‐invasiveness property, and the mechanism by which erinacine A inhibited aggressiveness remains poorly understood.

Colorectal cancer (CRC), an aggressive malignant disease with a poor prognosis, is the fourth leading cause of cancer‐related death in the industrialized world 13. A large body of evidence indicates CRC cells’ self‐sufficiency in growth signals, their ability to escape from apoptosis, and their tendency towards tissue invasion and metastasis 14. Moreover, actin reorganization has been recognized as a critical cellular response that influences the induction of apoptosis and the inhibition of cell migration triggered by dietary phytochemicals in colon cancer cells 15. Recently, the role of intracellular reactive oxygen species (ROS), the level of which is elevated in CRC and sensitive to oxidative damage, suggests that phenolic phytochemicals having antioxidant activity should short circuit the signalling events and eventually inhibit cancer cell proliferation 16. In the previous study, we identified a newer cytotoxic agent to be used against CRC 12. The survival curve showed that dietary *H. erinaceus* mycelium and its isolated diterpenoid derivative, erinacine A, had a dose‐dependent effect on the cytotoxicity *in vitro* and *in vivo*. However, little is known about the anti‐invasiveness, and the mechanism by which erinacine A possesses therapeutic potential for CRC invasion remains poorly understood. Further study aimed to investigate the objects of erinacine A's target proteins and to evaluate anti‐invasion potential involving the PI3K/mTOR/p70S6K signalling pathway.

In our aim to illustrate the molecular mechanisms underlying erinacine A–induced ROS production and the activation of the PI3K/mTOR/p70S6K signalling pathway in HCT‐116 CRC cells, we found that the protein profiles offered information leading to the discovery of specific biomarkers and the mechanism of *H. erinaceus* mycelium. In this study, we also found the inhibition effect of erinacine A on cell invasion, and we used two‐dimensional electrophoresis (2‐DE)‐based proteomic analysis to identify the proteins COFL1 and PROF1, potentially important molecular targets involved in the activation of the PI3K/mTOR/p70S6K signalling pathway and oxidative stress. Our results, further confirmed by the *N*‐acetyl cysteine (NAC) and specific inhibitors’ administration showed erinacine A's involvement in the PI3K/mTOR/p70S6K pathway and anti‐invasion, thereby showing COFL1 and PROF1, which potentially involved in anticancer mechanisms of erinacine A in the human HCT‐116 and DLD‐1 cells, previously had been implicated in the cancer‐related actin depolymerization pathway and CRC therapy 17. This study, with *in vitro* and *in vivo* pathological analysis, demonstrated that *H. erinaceus* mycelium and the native erinacine A could possess therapeutic potential for CRC invasion.

## Materials and methods

### *Hericium erinaceus* extracts and analysis of erinacine A

The fermentation process of *H. erinaceus* mycelia was performed and then cultivated, harvested, lyophilized, grounded to powders and was kept in a desiccator at room temperature. Fresh mycelium of *H. erinaceus* was extracted with ethanol. The extract was concentrated and fractionated by solvent partition between ethyl acetate and water. The proximate composition analysis was subjected to silica gel column chromatography according to the previous study 10, 12, 18; high‐performance liquid chromatography (HPLC) analysis of erinacine A was executed according to the previous study with minor modifications. The analytical column used was a COSMOSIL 5C18‐AR‐II (250 × 4.6 mm; particle size 5 μm, Nacalai USA, Inc., Kyoto, Japan). The retention time of erinacine A was approximately ~17.4 min at a flow rate of 1.0 ml/min. with a scanning UV wavelength at 340 nm. The 5 mg/kg erinacine A in the *H. erinaceus* extracted with 85% ethanol was confirmed and quantified by HPLC 10, 12. Chemical compounds studied in this article Erinacine A (PubChem CID: 10410568), as shown in Figure 1.

### Cell culture

The human HCT‐116 and DLD‐1 cells were purchased from the American Tissue Culture Collection (ATCC, Manassas, VA, USA). Cells were grown in DMEM supplemented with 10% foetal calf serum (Gibco, Grand Island, NY, USA), non‐essential amino acids, 1 mM sodium pyruvate and 1% antibiotics (100 units/ml of penicillin and 100 μg/ml of streptomycin). All experiments were performed in plastic tissue culture flasks, dishes or in microplates (Nunc, Naperville, Denmark). Incubation was carried out at 37°C in a humidified atmosphere of 5% CO_2_ and 95% air 19.

### Chemical reagents and antibodies

All culture materials were purchased from Gibco. 3‐(4,5‐dimethylthiazol‐2‐yl)‐2,5‐diphenyltetrazolium bromide (MTT), ROS scavenger (NAC), 2,7‐dichlorodihydrofluorescein diacetate (H_2_DCFDA), dihydroethidium (DHE), PI3K inhibitor (Wortmannin) and mTOR inhibitor (rapamycin) were purchased from Sigma‐Aldrich (St. Louis, MO, USA). Mouse monoclonal antibodies against cofilin (COFL1), Cofilin Ser^3^, profilin (PROF1) as well as hepatoma‐derived growth factor (HDGF) and nucleophosmin (NPM), and β‐actin and horseradish peroxidase‐linked anti‐rabbit or mouse IgG were purchased from Santa Cruz Biotechnology (Santa Cruz, CA, USA). Rabbit polyclonal antibodies against PI3K Ty^r458^, AKT Ser^473^, p70S6K Thr^389^ as well as ROCK1 (C8F7), LIMK2 Thr^505^ were purchased from Cell Signaling Technology (Beverly, MA, USA). SDS, NP‐40, sodium deoxycholate and protease inhibitor cocktails were purchased from Sigma‐Aldrich.

### Measurement of cell viability and reactive oxygen species

Cell viability, as previously reported by Annexin V–FITC/propidium iodide (Biosource International, Camarillo, California, USA), was used to quantify the percentage of cells undergoing apoptosis. The intracellular accumulation of ROS (O_2_^−^) was determined by using the fluorescent probes of H2DCFDA (2,7‐dichlorodihydrofluorescein diacetate) and the cells were washed prior to FACS analysis and Cell Quest software was used (Becton Dickenson, Franklin Lakes, NJ, USA). The results were presented as a percentage of the fluorescent intensity compared with the control sample. Data were analysed with CellQuest and WinMDI software. The apoptotic cells (V^+^/PI^−^) were measured by the fluorescence‐activated cell sorter analysis in a FACS analyser (Becton Dickinson). The data represented three independent experiments 19.

### Matrigel invasion and scratch assays

The Boyden chamber assay used for the analysis of tumour cell invasion is based on a chamber with two medium‐filled compartments. HCT‐116 cells were allowed to grow as discrete colonies as described above. Cells were collected by trypsin and suspended in serum‐free medium at 1 × 10^5^/ml. Cells (1 × 10^5^/ml) in serum‐free medium were added to an inner cup of the 48‐well Transwell chamber (Corning Life Sciences, Corning, NY, USA) that had been coated with 50 μl of Matrigel (BD Biosciences, Franklin Lakes, NJ, USA; 1:10 dilution in serum‐free medium). Medium supplemented with 10% serum or indicated agent was added to the outer cup. Cells were allowed to migrate for 24 hrs at 37°C in a humidified atmosphere containing 5% CO_2_. The membrane was fixed and stained with modified Giemsa stain (Sigma‐Aldrich). Cells on the lower side of the membrane were counted using a light microscope at 200× magnification. The number of cells that migrated to the lower side of the membrane was determined 19.

Scratch assays were performed by plating cells in a 6‐well culture dish. After the cells were allowed to attach and reach confluence, a scratch (4 mm) was made through the culture dish. The cells were washed twice with PBS (pH = 7) before their subsequent incubation with culture medium in the absence (control) or presence of erinacine A. Openlab v3.0.2 image analysis software (Improvision, Coventry, UK) was used to quantify the area progressively filled with cells over the period of the experiment 19.

### *In vivo* treatment and histopathological evaluation

These nude mice were performed on according to the modified previously, which were injected subcutaneously with HCT‐116 cells (10^6^ cells/0.2 ml) into the flanks of female athymic BALB/c‐*nu* mice (4–6 weeks old) 12. The control animals were treated daily with 0.1 ml DMSO (0.25%; i.p.), and the test animals were treated with erinacine A (1, 2, 5 mg/day; i.p.) for 5 days. Tumour volumes were monitored, removed and assayed. The tumour tissues were fixed in 10% buffered formalin, embedded by paraffin and cutted into 5‐μm‐thickness slides transversely and histopathological changes in the cells morphology were examined by light microscope (Olympus, Tokyo, Japan) at high power (200× magnification) for each slide. For immunohistochemical analysis, subcutaneous tumour specimens were incubated with monoclonal anti‐cofilin (COFL1 Ser^3^), profilin (PROF1) and AKT Ser^473^, p70S6K Thr^389^antibodies (Santa Cruz Biotechnology). The immunoreative cells (brown) were counted. The digital images were captured using a digital camera (Canon A640, Tokyo, Japan). The positive area and optical density (OD) of positive cells + (brown) were determined by measuring three randomly selected microscopic fields (400× magnification) for each slide. The Immunohistochemistry (IHC) index was defined as the average integral optical density (AIOD) (AIOD = positive area × OD/total area) 11.

### Second dimensional protein electrophoresis analysis of HCT‐116 cells proteomic profiles

The chemicals and reagents used for 2D gel electrophoresis were as described in our previous study 20. *Erinacine A*–treated and untreated HCT‐116 cells were harvested and protein extraction was carried out. Prior to 2D‐PAGE analysis, the protein concentration was measured using the Bradford assay with bovine serum albumin as the standard sample for normalization; following cell lysis, the total cell protein was precipitated by 10% trichloroacetate in acetone. Protein samples were suspended in rehydration solution and subjected to isoelectric focusing in 13‐cm, nonlinear, pH 3–10 immobilized‐gradient strips (Immobiline DryStrips; Amersham Biosciences, Uppsala, Sweden) in an Ettan IPGphor II apparatus (Amersham Biosciences). The second dimension electrophoresis was carried out using 10% SDS‐PAGE gels.

### In‐gel digestion and identification of peptide fingerprints with erinacine A treatment using MALDI‐TOF

Six pairs of silver‐stained 2D SDS‐PAGE gels in which total cell proteins had been resolved were scanned using ImageMaster 2D Platinum Software 6.0 (Amersham Biosciences). The protein profiles of each pair of silver‐stained gels were recorded and compared and then only the protein spots that differed by at least threefold among all six pairs of 2D gels were subjected to in‐gel digestion for matrix‐assisted laser desorption ionization time‐of‐flight/time‐of‐flight (MALDI‐TOF/TOF) mass spectrometric analysis. The gel pieces were then dehydrated and subjected to trypsin digestion. Mass spectra were acquired as the sum of the ion signals that were generated by irradiation of the target with a mean of 300 laser pulses using the FlexAnalysis system (Bruker‐Franzen Analytik, Bremen, Germany). Peptide fingerprints were selected in the mass range 700–4000 D, and then analysed by the Mascot software (http://www.matrixscience.com). A Mascot score with *P* < 0.05 was considered statistically significant as described in our previous study. MALDI‐TOF/TOF data were searched in‐house MASCOT software (ver 2.2.04). The protein identifications required detection of unique peptides, and proteins with more than two spectral counts were selected for further analysis. The peptide mass data of each spot were submitted to the SwissProt 100425 human species bioinformation stations using MASCOT search engines. Proteins identified with a higher MASCOT score in the bovine database than in the human database were considered as serum contamination and removed 20.

### Preparation of total cell extracts and immunoblot analyses

Cells were lysed with a buffer containing 1% NP‐40, 0.5% sodium deoxycholate, 0.1% SDS and a protease inhibitor mixture (phenylmethylsulfonyl fluoride, aprotinin and sodium orthovanadate). The total cell lysate (50 μg of protein) was separated by SDS‐PAGE (12% running, 4% stacking) and analysed by using the designated antibodies and the western‐light chemiluminescent detection system (Bio‐Rad, Hercules, CA, USA), as previously described 19.

### shRNA lentivirus transfection

HCT‐116 cells were infected with the designated lentiviral control, COFL1 and PROF1 plasmids by using shRNA expression of lentiviral Particles (Santa Cruz Biotechnology) and methods of confirming and applying the knockdown of genetic functions were established (Santa Cruz Biotechnology).

### Statistical analysis

Data were reported as the mean ± S.D. of three independent experiments and were analysed by one‐way anova. The data were analysed using the SAS software statistical package ‘SigmaPlot’, version 9.0 (SAS Institute Inc., Cary, NC, USA) 19.

## Results

### Anticancer effect of erinacine A–mediated reduction in HCT‐116 cell migration and invasion

**Figure 2:**
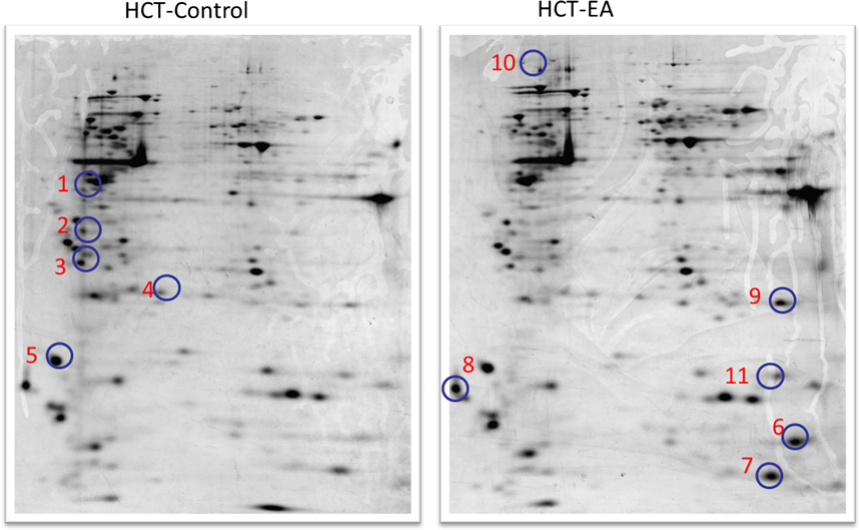
Representative two‐dimensional gel electrophoretograms of erinacine A treatment on HCT‐116 cells. Six pairs of cell protein extracts were evaluated, and a representative pair of the proteomic gel images from the erinacine A–treated and untreated groups are shown. Ten 15 protein spots with a threefold difference between both groups were subjected to MALDI‐TOF‐TOF analysis. Five protein spots with a significant decrease compared with the untreated group were encircled and annotated. Five spots with a significant increase in the erinacine A group were reported. The full names of these differentially displayed protein spots are listed in Table 1, respectively.

Our previous study demonstrated that *H. erinaceus* mycelium and its structural analogue, erinacine A, inhibit DLD‐1 proliferation and cell cycle distribution by activating the mTOR/NFκB and p21 pathways and allowing them to show that this activation is related to ROS generation 12. Based on these studies, by evaluating the apoptosis and anti‐invasion potential involving the signalling pathway, we assayed whether *H. erinaceus* mycelium provides substantial therapeutic advantages. Using the scratch‐wound assay, a continuous rapid movement was observed for HCT‐116 and DLD‐1 cells, but a resultant movement of a cell migration front was clearly evident at 24 hrs, where a highly confluent (90–100%) monolayer region gradually migrated into the cell‐free ‘scratch’ region. In the presence of erinacine A, migration was significantly inhibited after 12 and 24 hrs, whereas treatment with 30 μM erinacine A led to a virtually complete inhibition of cell migration (Fig. 1A). The Boyden chamber assay was used to determine the inhibitory effect of treatment with erinacine A on HCT‐116 and DLD‐1 cells invasiveness. The quantification of observations revealed also exhibited a significant anti‐invasive effect as compared to the control group, respectively (Fig. 1B), which supported the results obtained with the scratch‐wound assay in a dose–response relationship (Fig. 2). In addition, erinacine A initially increased the intracellular ROS production in HCT‐116 cells after 6 hrs, as compared to the control group (Fig. 1C). It was interesting to observe that, based on the cell viability assay, erinacine A treatment exhibited an inhibitory tumour growth effect on HCT‐116 cells (data not shown).

### Proteomic profiling of HCT116 cells treated with erinacine A

**Figure 3:**
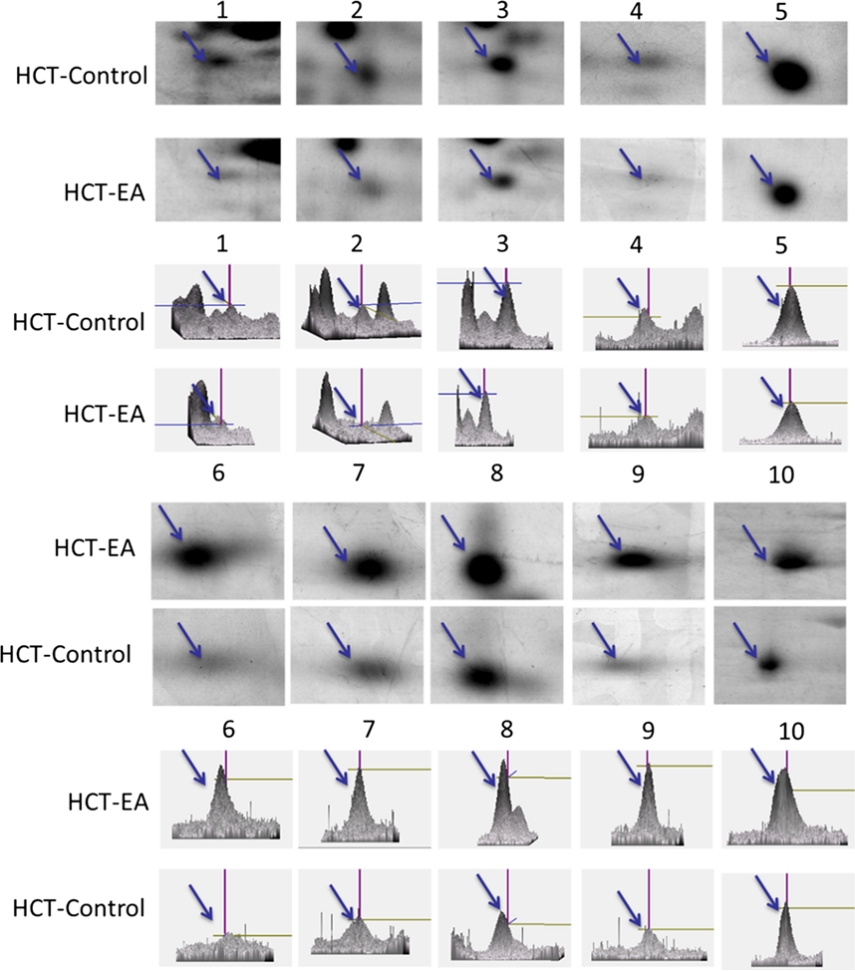
A close view of selected protein spots representing differentially expressed erinacine A–related proteins between erinacine A–untreated and treated HCT‐116. The proteins with expression compared with the control group are shown. The dot number corresponds to that in Table 1. Six pairs of cell protein extracts were evaluated, and the results shown were derived from one pair of experiments; four reproducible blots were performed in total.

To clarify the events of erinacine A–inhibited aggressive phenotype and stress responses, in our proteomics study of CRC HCT‐116, cell lysates were prepared and then more than 800 protein spots were visualized. Several differences were observed after erinacine A treatment in the silver‐stained 2D‐PAGE analysis. For the image analysis, protein expression profile gels (six pairs from the control and the erinacine A–treated groups) were compared using ImageMaster software. The expression level for the 10 total protein displays was at least threefold greater in the erinacine A–treated group than in the untreated control. Figure 2 illustrates the representative images of silver‐stained gels. The 10 spots that displayed a remarkable differential expression were chosen by numbered outlines. With erinacine A treatment, five protein spots showed a greater than threefold change in density (Fig. 3), whereas five proteins, down‐regulated consistently by treatment and exhibiting a greater than threefold increase in expression, were identified (Fig. 2A). These 10 spots were subjected to peptide fingerprint identification using MALDI‐TOF MS. Table 1, presenting the zoomed views of a representative gel's region, displays several differentially expressed oxidative stress‐related proteins on the target of erinacine A, which inhibits invasive action in HCT‐116. The statistical analyses were conducted as mean plus the standard deviation for the quantified differentially expressed proteins in 10 different proteins (Student's *t*‐test *P* < 0.01).

**Table 1:** Differentially expresses proteins during erinacines A treatment of U87 cells

Spot	Protein name	*Mr/PI*	Accession no	MASCOT score	Matched peptides
1.	Hepatoma‐derived growth factor	26/4.5	HDGF_HUMAN	108	10
2.	Tropomyosin alpha‐3 chain	32/4.5	TPM3_HUMAN	103	15
3.	Proteasome subunit alpha type‐5	26/4.5	PSA5_HUMAN	310	28
4.	Glutathione S‐transferase P	23/5.3	GSTP1_HUMAN	331	32
5.	Nucleophosmin	32/4.4	NPM_HUMAN	128	12
6.	Profilin‐1	15/9.3	PROF1_HUMAN	345	49
7.	Macrophage migration inhibitory factor	12/9.1	MIF_HUMAN	244	23
8.	Peroxiredoxin‐1	22/9.2	PRDX1_HUMAN	475	78
9.	Heat shock protein HSP 90‐beta	83/4.8	HS90B_HUMAN	832	71
10.	Cofilin‐1	18/9.1	COF1_HUMAN	127	13

### Validation of the differentially displayed proteins *in vitro* and *in vivo*

**Figure 4:**
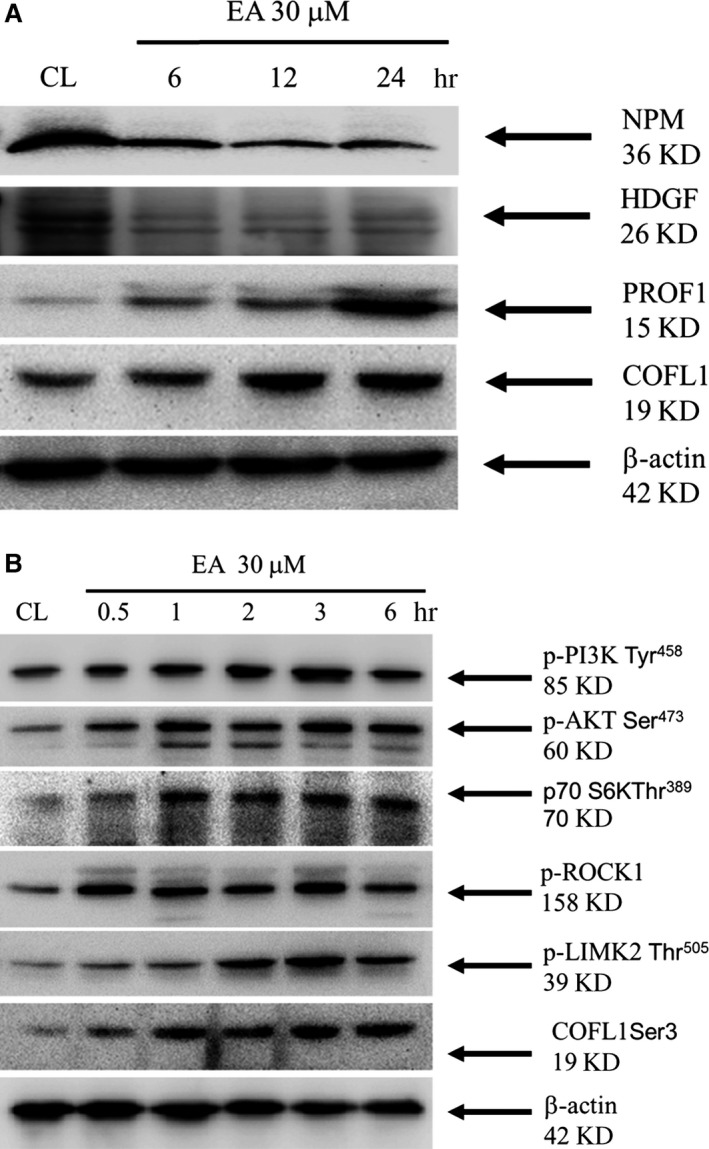
Validation of the identified differential proteins and activation of PI3K/mTOR/p70S6K and ROCK1/LIMK2/Cofilin pathways between erinacine A–untreated (CL) and treated (EA) in HCT‐116 cells. (**A**) Data presented in the Western blot are derived from a representative study, and comparisons of protein expression are calculated from three replicate experiments. The proteins with higher expression (PROF1 and COFL1) and with lower expression (NPM and HDGF) in erinacine A–treated cells are shown for the indicated time. (**B**) The effect of erinacine A on protein levels of phosphorylated PI3K, AKT, p70S6K, ROCK1, LIMK2, Cofilin and β‐actin were detected with the indicated antibodies.

Spots 1, 5, 6 and 10 were subsequently identified using 2D proteomic analysis, including HDGF and NPM as well as cofilin (COFL1) and profilin (PROF1), which may mediate oxidative stress and the aggressive effects of erinacine A in HCT‐116 cells (Table 1). To confirm the association of erinacine A in the interaction between pharmaceutical intervention and redox imbalance potential from 2D proteomic analyses, we identified these proteins for further study using Western blotting assays. As shown in Fig. 4A, we selected these proteins for further study using Western blotting procedures after treatment with erinacine A. The results are consistent with the proteomic results, demonstrating reduced expression of HDGF and NPM. In general, erinacine A induction resulted in a significant level of COFL1 and PROF1 (Fig. 4A).

### Effects of erinacine A on the up‐regulation of COFL1, PROF1 and the PI3K/AKT/p70S6K/ROCK1/LIMK2/Cofilin‐signalling mechanism as well as ROS production

It has been reported that the second‐messenger properties of ROS are believed to activate signalling pathways by activating tyrosine kinases and tyrosine phosphatases. MAP kinases, Rho‐GTPase and PI3K/AKT/p70S6K/MAPK are the main contributors to targeted therapy‐induced CRC toxicity, causing phytochemicals’ antioxidant activity to inhibit cancer cell proliferation 21. Combined with this finding is the fact that increased ROS production induced by erinacine A 19 is often accompanied by the activation of uncoupling proteins as a mechanism that decreases cell growth and the aggressive status of HCT‐116 cells. As shown in Table 2, HCT‐116 cells were subjected to erinacine A and then co‐treated with ROS scavenger NAC, PI3K inhibitor Wort and mTOR inhibitor Rapa, as well as Lentiviral control, shRNA COFL1 and shRNA PROF1. These treatments significantly reversed the erinacine A–induced cell death and then decreased the erinacine A–induced cells migration and invasion at 24 hrs, respectively (*P < 0.01). The results showed the addition of erinacine A, Lenti shRNA PROF1 and COFL1 were confirmed that the ROS generation of erinacine A on HCT‐116 cells is mediated *via* the PI3K/AKT/p70S6K signalling pathways and up‐regulation of PROF1 and COFL1. Previous studies focused on the actin regulatory protein cofilin; it showed that increased ROS production induced apoptosis by the phosphorylation of ROCK/LIMK. This was often followed by the inactivation of tumour metastasis as a mechanism decreasing mitochondrial function 16, 17, 22. We detected two proteins, COFL1 and PROF1, which previously had been involved in the apoptosis process and tumour suppression 23, 24. We set out to further clarify the molecular mechanisms underlying the erinacine A–mediated effects of erinacine A–mediated ROS production and the beginning of signalling pathways in human CRC. We observed that stimulation with erinacine A significantly increased the total lysate protein with the phosphorylation of PI3K, AKT, p70S6K, ROCK1, LIMK2, Cofilin at ser3, which was compared with the control (CL) a half hour after the indicated dose (Fig. 4B).

**Table 2:** Effects of the kinase inhibitor on the erinacines A induction associated with cell death and aggressive status in HCT‐116 cells

	% of cell death	Migration (%)	Cell invasion (%)
Control	14	100	100
Erinacines A	58	10	40
Erinacines A NAC	18	82	80
Erinacines A Wort	25	54	62
Erinacines A Rapa	28	46	52
Erinacines A shGFP	25	54	62
Erinacines A shCOFLI	28	46	52
Erinacines A shPROF1	28	46	52

### COFL1 and PROF1 were involved in the regulation of the PI3K/mTOR/p70S6K signalling pathways as well as reactive oxygen species on apoptosis and invasion by erinacine A in HCT‐116 and DLD‐1 cells

**Figure 5:**
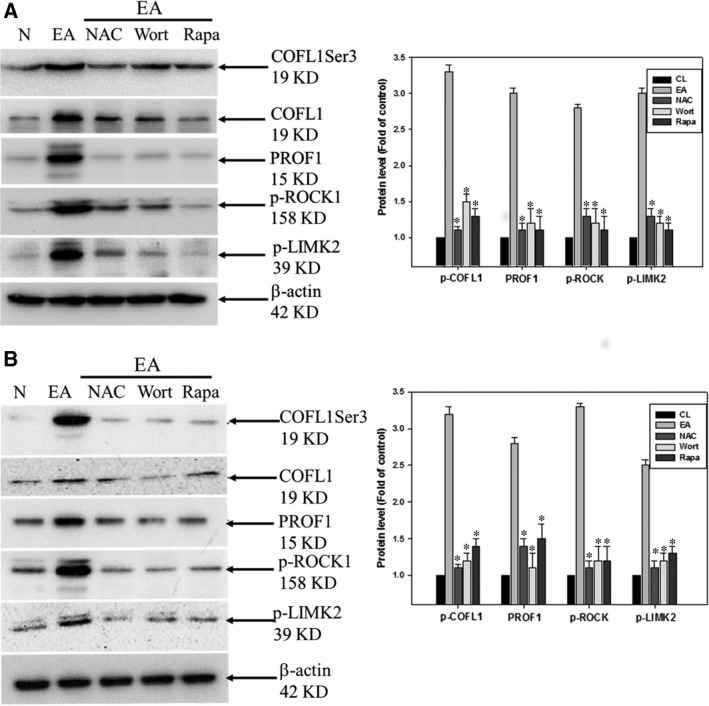
Effects of the kinase inhibitors blocking erinacine A–induced activation of ROCK1/LIMK2/Cofilin pathway–related proteins. Kinase inhibitors were treated either with or without erinacine A in HCT‐116 (**A**) and DLD‐1 cells (**B**) after 1 hr. All cell lysates were prepared and subjected to Western blot analysis. Protein levels of phosphorylated ROCK1, LIMK2 and Cofilin as well as PROF1, COFL1 and β‐actin were detected with the indicated antibodies. Protein levels were quantified by densitometric analysis, with the control being set at 100%; actin was used for equal loading as the control. Data are presented as means ± S.D. of three independent experiments. *P < 0.05, when compared with the untreated control group.

**Figure 6:**
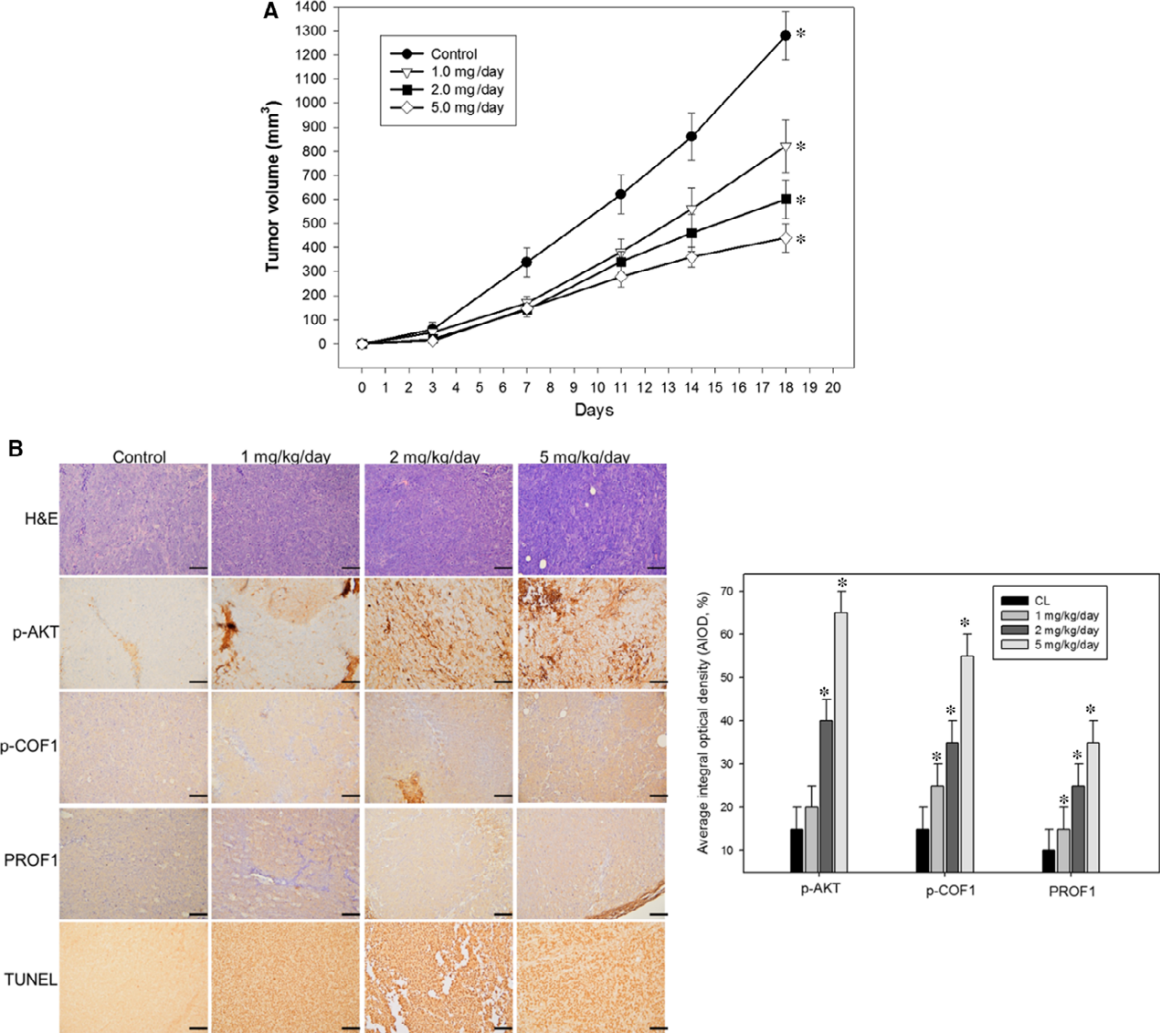
Immunohistochemical staining for indicated proteins expression in tumour sections was determined. (**A**) Time‐courses effect of erinacine A on the growth of HCT‐116 cells xenograft was evaluated by determining the tumours volume. (**B**) H&E staining revealed similar s.c. tumour morphology among groups of tumours. p‐AKT, PROF1 and COFL1 staining showed expressed tumour cells treated with erinacine A. TUNEL staining revealed significantly greater apoptosis in response to erinacine A in tumours. Quantitative immunohistochemical proteins PROF1 and COFL1 were evaluated by average integrated optical density (AIOD). The positive stained area was evaluated from three randomly selected observation fields of each brain section. Data were expressed as mean ± S.D. (*n* = 6/group). *P < 0.05, compared with the control group, magnification ×400.

Our results clearly confirmed that erinacine A treatment mediated apoptosis and anti‐aggressive pathways in HCT‐116 cells through the ROS trigger and that upstream PI3K/mTOR/p70S6K was associated with the ROCK1/LIMK2/Cofilin pathways. To further determine whether erinacine A induced phosphorylation of the ROCK1/LIMK2/Cofilin pathway and COFL1 and PROF1 levels in human HCT‐116 and DLD‐1 cells, we investigated whether the effects of kinase inhibitors caused resistance to erinacine A–induced apoptosis and inhibition invasiveness. We conducted a basic study to demonstrate the novel role of COFL1 and PROF1 in erinacine A's actions. As shown in Figure 5, NAC and Wort almost blocked the erinacine A–induced levels of COFL1 and PROF1 as well as the phosphorylation of ROCK1/LIMK2/Cofilin, as compared to the sole use of erinacine A treatment for 3 hrs. Taken together, the results and the data showed that COFL1 and PROF1 expression in HCT‐116 and DLD‐1 cells is essential to implication in oxidative stress and PI3K/mTOR/p70S6K, signalling that erinacine A inhibited cell invasion and tumour growth. In addition, HCT‐116 cells’ xenograft growth was inhibited *in vivo* by erinacine A for 3 weeks as modified in our previous study. Differentially displayed proteins were assayed from all tumour sections and validated; we then used further examination employing immunohistochemistry assays after cell xenograft and erinacine A treatment to identify COFL1 and PROF1 proteins. As shown in Figure 6, HCT‐116 xenograft mice with erinacine A demonstrated that intraperitoneal injections of erinacine A (1–5 mg/kg/day) treatment significantly inhibited the tumours volume and increased COFL1 and PROF1 significantly induced protein expression in the tissue samples. In particular, erinacine A–induced apoptosis damage resulted in significant phosphorylation of the cofilin level at ser3. These results are consistent with the proteomic results, indicating that a proteomic differential display model is applicable when assessing erinacine A–inhibited HCT‐116 cells.

## Discussion

Colorectal cancer is a sequential multistage process consisting of tumour initiation, tumour promotion and tumour metastasis 14. Despite intense efforts to develop treatments, effective agents have not yet been found. Chemopreventive compounds in foods are a potential source of bioactive safe compounds for cancer chemoprevention and suppression of metastasis by a variety of mechanisms, including consequences of ROS stress that damage DNA and activation of signalling pathways, and expression of proteins involved in the inhibition of cancer cell metastasis 25, 26. *Hericium erinaceus* has long been used for its beneficial health properties, which are reported to be medicinal with the effect of inhibiting the metastasis of cancer cells 27. Previous studies have shown the ability of the fruit body extracts of *H. erinaceus* to induce apoptosis using CT‐26 murine colon carcinoma cells as an indicator of the inhibition of cell migration and invasion 28. The same extracts reduced the expression of the matrix metalloproteinases MMP‐2 and MMP‐9 as well as the urokinase‐type plasminogen activator, which led to reduced phosphorylation of MAPK 29. However, the biochemical targets of *H. erinaceus* mycelium isolated erinacine A on the anticarcinogenic properties of CRC cells and how the signal cascades become activated have not been comprehensively recognized. Most interesting in our observations was the effect that erinacine A had on HCT‐116 and DLD‐1 cells migration and invasiveness, as it significantly lowered the cells’ aggressive status (Fig. 1). Here, the results strongly suggest an essential role for the PI3K/AKT and mTOR pathways during the execution of apoptosis and anti‐invasion by using naturally extracted erinacine A (Fig. 4B, Table 2). This study elucidated the mechanism of the observed inhibition of metastasis of CRC cells, suggesting that inhibition of metastasis is related to the composition of *H. erinaceus* mycelium.

Many studies have shown that cellular mechanisms contribute to the overall cancer prevention effects of these dietary phytochemicals 19, 26, 30. Our previous studies have suggested that isolated erinacine A, a diterpenoid compound, causes ROS generation, which possibly plays an important role in mitotic arrest and tumour growth inhibition in human CRC cells 19. In this study, we have shown how the method of erinacine A treatment may be used to inhibit tumour invasion of HCT‐116 cells through ROS increment and activation of the PI3K/mTOR/p70S6K signalling pathway. Previous studies have demonstrated that transient changes in the overproduction of ROS can affect the activity of the signal transduction pathways, leading to either cell cycle arrest, or to apoptosis and invasiveness, with respect to the dosage and duration of ROS and also the type of cell caused by phenolic phytochemicals 15, 16, 21. This study demonstrates that HCT‐116 and DLD‐1 cells treated with erinacine A shows oxidative alterations in terms of signalling transduction events and ROS overloading that result in therapeutic effects on cancer invasion (Fig. 1C, Table 2) 12. To identify molecular targets related to erinacine A effects, we used a 2‐DE‐based proteomic analysis to identify protein expression profiles on erinacine A treatment mechanisms (Fig. 2). Cofilin (COFL1) and profilin (PROF1) were more abundant in erinacine A induction, whereas HDGF and NPM were at a lower level *in vitro* and *in vivo* (Figs 3 and 6). Recent studies have suggested that HDGF is a novel multifunctional growth factor that has had major implications in biological processes such as tumour proliferation, invasion, angiogenesis and apoptosis, and that HDGF might be a potential therapeutic target for human CRC 31, 32. Dysregulation of NPM in numerous solid and haematological malignancies has been found to support its oncogenic role and prognostic markers in colon and rectal cancer development 33. It is interesting that several proteins such as PROF1 and COFL1 are regarded as having tumour suppressor‐ and tumour invasiveness‐related proteins 23, 24. A recent study has shown that PROF1, a component of G‐actin‐binding protein, acts as a tumour suppressor gene that adds ATP‐bound G‐actin to the barbed ends of growing cytoskeleton filaments, correlating Pfn1 expression with motility and invasiveness of tumour cells 24. In addition, cofilin has a central role in controlling actin dynamics, by regulating actin polymerization and actin depolymerization through its severing activity, as well as by involving DNA damage response for cancer therapy 34. The actin depolymerization pathway has been recognized as a critical cellular response that controls the apoptosis and inhibition of cell migration triggered in prostate, breast and colon cancer cells through ROCK1/LIMK2/Cofilin cascades. Based on the proteomic differential displays of HCT‐116 cells 22, 35, our results showed the important finding that these activation effects result from a downstream gene of PROF1 and COFL1 expression and the phosphorylation of the PI3K/mTOR/p70S6K and ROCK1/LIMK2/Cofilin pathways as well as the execution of apoptosis and anti‐invasiveness by erinacine A (Figs 5 and 6). The aim of this study was to clarify the mechanisms of CRC invasion inhibition and activation of the PI3K/AKT/p70S6K and actin depolymerization pathways, as well as the generation of ROS induced by erinacine A in human HCT‐116 cells. It suggested that the generation of ROS as well as the PI3K/AKT/p70S6K pathway to promote apoptosis and decreased invasiveness could be partly caused by the PROF1 and COFL1 expression by erinacine A. Thus, *H. erinaceus* mycelium represents a novel chemotherapeutic agent worth continued investigation. To validate these findings in particular, further study using other differential proteins is needed to determine whether there are mediated cytoskeleton actions.

**Figure 7:**
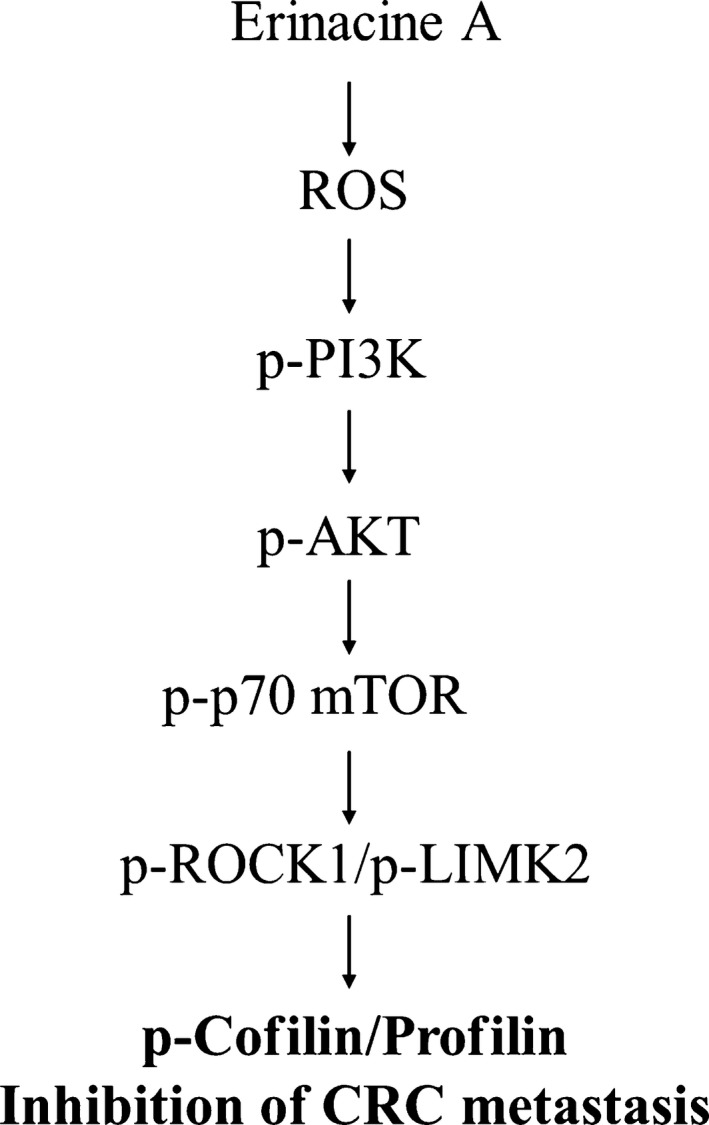
Schematic presentation of the signalling pathways involved in erinacine A–inhibited cell invasion in HCT‐116 cells. The effect of erinacine A on production of ROS activates phosphorylation of PI3K/AKT/mTOR/p70S6K and ROCK1/LIMK2/Cofilin pathways, which induces the COFL1 and PROF1 up‐regulation and increases inhibition of CRC metastasis.

In conclusion, on the basis of proteomic differential proteins, we suggest that the up‐regulation of PROF1 and COFL1 expression of erinacine A is mediated by actin alterations, which are preceded by the ROS as well as the PI3K/AKT/p70S6K and ROCK1/LIMK2/Cofilin pathway, with an induction of ROS (Fig. 7). This study is potentially interesting with regard to the antitumour effect of erinacine A found in the *H. erinaceus* mycelium as it relates to the development of novel chemotherapeutic approaches in the treatment of malignant CRC and cancer development in general.

## Conflict of interest

The authors confirm that there are no conflicts of interest.
